# Considerations and Perspectives on Interventions to Improve Social Cognition in Adolescents With ASD Without ID: Involving Parents in Social Skills Groups

**DOI:** 10.3389/fpsyt.2021.629761

**Published:** 2021-05-28

**Authors:** Delphine Vuattoux, Deborah Castiglia, Nadia Chabane

**Affiliations:** Division of Autism Spectrum Disorders and Related Conditions, Department of Psychiatry, Lausanne University Hospital and University of Lausanne, Lausanne, Switzerland

**Keywords:** Autism Spectrum Disorder without intellectual disability, adolescents, parents, evidence-based interventions, social skills groups

## Abstract

Improving social cognition and social skills is a challenge faced by individuals with Autism Spectrum Disorder without Intellectual Disability at any age. This process is particularly critical during late adolescence (15–18 years), a developmental phase generally characterized by rich social experiences that usually foster the development of friendships. Nevertheless, for youth with ASD, lingering difficulties in social cognition often hinder their ability to generate responses considered socially appropriated. These social deficits can contribute to isolation that has a detrimental effect on mental health. In adulthood, deficits of social skills are strongly associated with an overall lack of support, characterized by a failure to integrate into the labor market, a high unemployment rate, social isolation, and a higher suicide rate. In clinical settings, social skills groups are well-established therapeutic means to improve social cognition and social skills. Nevertheless, these interventions vary greatly regarding their objectives, contents and duration. Moreover, few have been validated and replicated by research. Our aim is to bring certain perspectives to a type of intervention that are widely used in care settings. After reviewing its positive aspects for increasing social cognition, and its limitations, we will discuss strategies to facilitate the generalization of social skills in an ecological context. In particular, we will base our reflection on our clinical experience and on our current project to adapt the PEERS model for adolescents into French. We will consider the current trend of involving parents as “social coaches” to generalize the social knowledge acquired in the social skills groups.

## Introduction

Autism Spectrum Disorder (ASD) is a neurodevelopmental disorder characterized by social communication deficits with the presence of stereotypical behaviors, sensory atypicalities, and restricted interests. The clinical presentations of ASD are heterogeneous, with various levels of intellectual and motor functioning and verbal skills (1). ASD is often associated with comorbid disorders such as anxiety or ADHD that have a significant impact on a person's functioning and, consequently, on their quality of life (2–4). ASD's prevalence globally increases and is now estimated at 18.5/1,000 (i.e., 1/54) in children of 8 years of age in the United States (5, 6).

In the case of individuals with ASD without intellectual disabilities (ID), it appears that improving social skills has long been considered as a main challenge throughout their development, particularly during adolescence and the transition to adulthood (7). Indeed, this critical period is characterized by a phase generally rich in social experiences that foster the development of friendly relationships with peers (8–10). These naturally learned behaviors and attitudes contribute to acceptance by peers and usually avoid marginalization. For adolescents with ASD without ID, difficulties often persist in social communication. This includes, for example, proposing topics for discussion or repetitive conversations based on their specific interests not always shared by peers (11, 12). Difficulties in the acquisition of social skills in ASD have been linked to deficits in social cognition. Based on the cognitive and emotional processes that underlie the understanding and prediction of others' behaviors, social cognition is a concept that encompasses several skills, ranging from basic recognition of emotions to more complex processes such as perspective taking, also known as theory of mind (12–14). These cognitive, affective, and behavioral skills form the basis of social skills and thus social behavior. Deficits in social cognition impact social skills and interfere with the production of responses considered socially appropriate. In a neurotypical world, these social challenges are, at any age, an isolating and exclusion factor from peers (15). Rejection also affects ASD youth's mental health and is a risk factor for developing depression or anxiety disorders (7, 10, 16). These difficulties continue in the access to employment in adulthood (17, 18). Social relationships remain a major factor in job rejection and a source of stress for workers with autism (19). Deficit of social skills are accompanied by an overall lack of support characterized by a failure to integrate into the labor market and a high unemployment rate (17, 19, 20). In fact, studies on adults with ASD without ID report psychiatric co-morbidities and increased social isolation with a higher suicide rate for this population (21).

In recent years, researchers and clinicians have focused on the development of interventions aimed at supporting youth with ASD without ID to better understand social situations and respond with appropriate social behaviors (22). To this end, over the past two decades, social skills groups emerged as a natural attempt to extend learning in an individual setting by grouping participants with common difficulties. Nevertheless, this common practice in health care settings has led to very variable support in terms of objectives, content and duration. For adolescents, the contribution of parents or other significant caregivers as “social coaches” and generalization agents was considered later (23, 24). Moreover, perceptions about people with autism are changing. Under a new vision of neurodiversity, their differences are beginning to be increasingly recognized as strengths rather than the sum of social deficits. We need then to look at these groups with new perspectives. The feedbacks of people with autism and their families appear to be an essential prerequisite for improving and redefine them. In this paper, our objective is, first of all, to review the elements validating the effectiveness of social skills groups and the limits to consider. We will then discuss on the active contribution of parents. Finally, we will conclude on new reflexions on these groups to best meet the current needs and expectations of young people with ASD.

## Social Skills Groups for Adolescents: Known Benefits and Limitations

First, how a social intervention can be considered “efficient”? In social skill group, we speak of effectiveness, when the objectives of acquiring new social skills are achieved (e.g., the adolescent can start and follow a reciprocal conversation with peers). Generalization refers to new skills that are reused appropriately outside the group in more natural contexts. One of the most important benefits of participating in a social skill group is the improvement of social knowledge and the quality of friendship relationships (25). Then, participation in a social skill group can also bring benefits in terms of improved emotional skills and social communication (26). Young people would have more opportunities to spend time with friends and would demonstrate a reduction in inappropriate social behaviors in favor of more adapted social behaviors (7, 27). Finally, this type of intervention is generally appreciated by participants for the sense of belonging and mutual support that the group provides in experiencing positive social interactions in a secure setting (28). Despite their popularity in clinical settings, the overall benefits of these interventions remain modest, with a small number of meta-analyses reporting improvements in social skills with average overall effect sizes (25, 29). In addition, a significant portion of validation studies has focused on evidence-based practices for preschool-age children and elementary school-age children (15, 30). Only a minority of the randomized controlled trials (RCT) identified in the most recent meta-analyses targeted social skills groups for adolescents (25, 29).

It is important to recognize that social skills are complex, context-related, and therefore subject to variation among different informants who observe change and on the instruments used to measure it (29, 31, 32). Most of the measures gathered in the literature are based on pre- and post-test questionnaires filled out by parents, adolescents, and in some cases by teachers and external observers (33). A high level of variability is then encountered in the results. The parental questionnaires most commonly used in programs are the Social Responsiveness Scale (SRS) and the Social Skills Rating System (SSRS) (34). The meta-analysis by Gates et al. (29) highlights a significant self-reported effect among youth in the overall improvement of their skills while parents do not report the same progress. This discrepancy in outcomes can be attributed to adolescents' bias to overestimate their skills (35). This effect would be enhanced by their sense of satisfaction after group participation (29, 33). In addition, the authors call for caution on outcomes. Indeed, a distinction must be drawn between “social knowledge” and “social performance,” two measures that are often analyzed conjointly (35, 36). When this distinction is taken into account, the results indicate a significant improvement in social knowledge among adolescents, but no benefits in terms of overall social performance (25, 29, 37).

Some other limitations about the social skills groups' effectiveness study must also be taken into account. Social skills groups are also heterogeneous in terms of curriculum content. Some intervention formats present the content and the behaviors to be adopted with a structured didactic approach and behavior rehearsals, designed as “social knowledge training,” while others, known as “social performance training” will rather focus into the experimentation of positive exchanges, where the skills are implicitly taught (29, 33, 38). Of the 25 manualized interventions identified by Hall et al. (39), only five programs offer an effectiveness study and only three consider generalization aspects. This is the case of Skillstreaming (40) aimed at teaching emotional and prosocial skills for adolescent with common social needs, Social Competence Intervention, a curriculum that combines cognitive-behavioral and applied behavior analysis principals to enhance specific social competences (27) and Program for the Education and Enrichment of Relational Skills, (PEERS) which focuses on improving friendship for Adolescents with ASD without ID (41). This latter program, with the most research published, will be discussed below.

For adolescents with ASD without ID, transferring new knowledge of social rules into various contexts and maintaining it over time remains a challenge (29, 37, 42). To date, few studies include follow-up measures for groups of adolescents with ASD without ID (24), especially since research findings are inconsistent in measuring generalization and skill maintenance (43).

## Involvement of Parents as Coaches and Generalization Agents

Clinicians observe that one of the most recurring requests from parents of adolescents with ASD without ID is precisely to target social skills work. They are very often worried about the social gap between their child and peers. Indeed, the differences experienced by adolescents can lead to a drop in self-esteem, anxiety, and depressive symptoms or manipulating conducts for the young person's social entourage (e.g., bullying). A recent review has identified the most effective components to generalize the skills acquired through participation in a social skills group (44). It appears that the individualization of objectives and the constant links with the adolescent's natural environment are precisely significant predictors of the effectiveness of these interventions. The presence of typical peers, the regular practice of the skills learned alongside them and the active involvement of parents also help to improve their effectiveness. Indeed, social skills groups that include groups of parents are those that obtain the most promising results with larger effect sizes (32).

For this reason, some programs involve parents as active support agents in the intervention. This is the case of PEERS, that our team has translated into French and is currently experimenting in a French-speaking context (French-speaking Switzerland) with adolescents with ASD without ID aged 15–18. PEERS is based on the joint involvement of adolescents and their parents with the common goal of learning and developing friendships (41). It emerges as one of the most research-validated manualized programs that has been replicated in different cultural backgrounds with a durability of these gains over time (39). Indeed, follow-up measures over 1–5 years have shown that social skills were maintained and improved, including social responsiveness, better social knowledge, and a higher level of social reciprocity with an increased number of get together with peers (24, 45). The maintenance of its effects could be explained by the participation of the parents who would have acted as agents of reinforcement and generalization of the acquired knowledge (24). This program includes 14 weekly 90-min social skills training sessions for adolescents with ASD without ID, while their parents attend parallel coaching sessions to teach them how to help their child to generalize these skills. In small groups (6–8 participants), the teaching of adolescents is based on learning practical rules governing social codes and skills that are ecologically valid, i.e., applicable in different contexts of adolescents' daily lives (e.g., developing friendships, knowing the rules of reciprocal communication, the etiquette of social networks and electronic communications, dealing with conflict and rejection, etc.). It is complemented by demonstrations of appropriate or bad social behavior models (video-modeling), role-playing and exercises for which adolescents receive feedback from facilitators and peers. The youth are given small homework assignments, which present gradual challenges to practice and generalize what they have learned between sessions. These cognitive-behavioral components are the ones that have been mainly found to be the most effective way to improve social skills (25, 46, 47).

During this time, parents work together on the same topic as their child. Another facilitator begins by talking with them about their child's successes and challenges related to the previous session, raising any difficulties in completing homework assignments, and finding new ways to accomplish them successfully. These sessions are not seen here as classic support sessions such as a support group because their objective is clearly to provide parents with tools related to a social theme (e.g., finding an appropriate group of friends, and managing rumors). Nevertheless, the atmosphere is generally warm between parents who share their experiences and are invited to propose solutions to the other participants. At the end of each session, teenagers and parents meet to agree on their roles and tasks for the next session. Outside of the sessions, parents take over to support the homework assigned to their child in order to repeat these teachings in a natural social context and to continue to generalize the skills discussed.

PEERS combines a didactic approach and social rehearsal. Its originality lies particularly in the involvement of parents. It appears that general participation in a psychoeducational parenting skills training program has a positive impact on their children's development as well as on their own well-being (48). In the PEERS model, their involvement can give them a sense of control over the difficulties they face and reduce significantly their stress (49). PEERS has been validated and replicated in other cultural backgrounds as Israel or Asian countries (50–53). In Europe, a Dutch team is currently conducting a RCT to compare their results with those of an active control group (54). At this stage, our experience remains very modest but our goal is to adapt it in a francophone RCT. Nevertheless, our preliminary observations are in line with results of larger samples, particularly in terms of increasing knowledge of social conventions. Overall, adolescents and their parents shared positive experiences with the program and the relevance of the new skills learned. As Mirzaei et al. (44) point out, active parental involvement thus brings an added value between what is learned in the rather limited context of the social skills group and the adolescent's life with peers and family in his or her daily life. The fact that parents are equipped and have access to the same lessons and content as their children, allows them to go beyond what is taught in a 14-session program.

## Going Further: Perspectives

With 20 years of hindsight in the field of social skills groups, researchers, and clinicians in all countries are now facing several observations but also questions to ensure that this type of intervention continues to gain in effectiveness. We will end here by addressing some practical aspects and raising more general questions.

First of all, due to the complexity of social skills, the characteristics of each participant and the variability of the available outcomes, the measurement of the effectiveness of social skills programs still needs to be improved. While self-report questionnaires remain an effective measure for improvements in social knowledge (32), more refined tools are needed to objectively measure adolescents' progress, especially since the questionnaires used have not been developed to specifically assess social skill groups (33). A valuable measure would be the involvement in programs of trained blind observers who could thus assess the ability of the participants to generalize their learning in different ecological settings (33, 55). However, access to and training of these observers involves a significant financial and time investment (24). In a recent study, Keifer et al. (31) support the importance of using different types of measures to observe changes in social cognition and social behavior. They point out that the general concept of social cognition needs to be further decomposed with the less studied measure of implicit social cognition defined by “processes that are fast, spontaneous, and primarily unconscious” (56). Yet, it is precisely these processes that would predict outcomes on social awareness and are observed through changes in young people's social behavior. The authors combined electrophysiological measures with other types of data such as the number of creative solutions proposed or the reaction time taken to solve a problematic social situation. Keifer et al. (31) recommend that it is therefore crucial that implicit social cognition be targeted by future interventions but also better measured as a marker of change in social skills.

Second, adolescents' motivation to participate in this type of program is an important question and may differ from their parents' motivation. Parents see or anticipate their children's difficulties, while teens do not necessarily see the benefits and claim their right to a certain social singularity. Their reticence is often explained by years of negative experiences that have led them to withdraw from the social world or to apprehend it through internalized or externalized disorders. It is important that adolescents feel fully free to accept or refuse to participate in a group despite certain well-intentioned pressures from those around them. Here, the fact that their parents are actively involved and block this time to support them in a concrete way may be a facilitating factor. Joint participation in the group can then be more easily accepted as a joint attempt to find solutions rather than another “extra-curricular” activity that rests solely on the adolescent's shoulders. It should be emphasized that this transition period to adulthood is often accompanied by a high level of anxiety experienced at the academic level (revisions, exams and preparation for team work) in addition to being associated with all the decisive questions of school and career orientation.

These questions about motivation and improvement of the effectiveness social skills groups finally lead us to take a step back and ask about the social objectives targeted solutions that we propose to try to overcome the “core deficits” of ASD. At present, the questions of camouflage, defined as behaviors used to hide or mask “autistic” aspects of one's personality, and social compensation strategies are more and more reported by adolescents and adults with ASD, who very generally name the exhaustion that the application of social rules engenders on their mental health (57). More than ever, it is a matter of working with autistic individuals themselves to determine their expectations, their needs and jointly establish the best tools to best navigate the complexities of the social world while recognizing their singularity and the right to neurodiversity. Bottema-Beutel et al. (37) thus warn of the risk that participation in a social skills group restricts the authenticity of its participants without diminishing the risks of stigmatization.

In a recent editorial on the issue of camouflage, Fombonne (58) “note[s] incidentally that most behaviors discussed in camouflage commentaries are precisely those skills that are actively targeted for scaffolding in social skill treatment interventions” (p.737). This perspective on the issue of teaching neurotypical social rules leads to the idea of continuing to improve the quality of our social skills groups by tailoring its objectives to the particular needs of its participants (27, 44). One proposal would be to revisit the experiences of those who have participated in these groups. In this way, qualitative research could clarify in greater detail how their participation met their needs or if group participation had contributed to feeling more stigmatized and anxious.

These ideas of close partnership have been taken up in different countries such as the United Kingdom, which encourages people with ASD to be active and paid consultants to help clinical teams to adapt medical interventions to their particular needs (59). In the social skills groups, participating youth and their parents could then become consultants whose experience would be valuable in improving the design of these groups, identifying the components that have been most helpful to them and the variables that remain impediments. Their subsequent participation as “mentors” and their testimonials about their progress after participating in such a group could also be a form of mentoring that would encourage their younger peers and their parents.

To conclude, social skills groups constantly lead us to question our tools. More broadly, they also lead us to reflect on our social rules, the behaviors defined by the group as socially appropriate and those that, on the contrary, are a source of rejection. We must then discuss together the bases governing life in the community in order to facilitate exchanges and to better understand behaviors that autistic and neurotypical people may each consider as “bizarre.” What are the social rules considered indispensable by all and which “battles” should we prioritize? On which rules should society be more tolerant and inclusive? These are all ethical questions that deserve to be tackled together and should guide the foundations of the implementation of future social skills groups.

## Author Contributions

DV and DC reviewed the available evidence and took the lead in writing the manuscript. NC provided critical feedback. All authors agree with the content of this manuscript.

## Conflict of Interest

The authors declare that the research was conducted in the absence of any commercial or financial relationships that could be construed as a potential conflict of interest.

## References

[1] American Psychiatric Association. Diagnostic and Statistical Manual of Mental Disorders. 5th edn. Washington, DC (2013).

[2] Moss P Mandy W Howlin P. Child and adult factors related to quality of life in adults with autism. J Autism Dev Disord. (2017) 47:1830–7. 10.1007/s10803-017-3105-5 28343343 PMC5432629

[3] Khanna R Jariwala-Parikh K West-Strum D Mahabaleshwarkar R. Health-related quality of life and its determinants among adults with autism. Res Autism Spectrum Disord. (2014) 8:157–67. 10.1016/j.rasd.2013.11.00332191120

[4] Tobin MC Drager KDR Richardson LF. A systematic review of social participation for adults with autism spectrum disorders: support, social functioning, and quality of life. Res Autism Spectrum Disord. (2014) 8:214–29. 10.1016/j.rasd.2013.12.002

[5] Maenner MJ Shaw KA Baio J Washington A Patrick M DiRienzo M. Prevalence of autism spectrum disorder among children aged 8 years - autism and developmental disabilities monitoring network, 11 sites, United States, 2016. Morb Mortal Weekly Rep. (2020) 69:1–12. 10.15585/mmwr.ss6904a1 32214087 PMC7119644

[6] Chiarotti F Venerosi A. Epidemiology of Autism Spectrum Disorders: A Review of Worldwide Prevalence Estimates Since 2014. (2020) 10:274. PubMed PMID: 10.3390/brainsci10050274 32370097 PMC7288022

[7] Volkmar FR Reichow B McPartland JC. Autism spectrum disorder in adolescents and adults: an introduction. In: Volkmar FR Reichow B McPartland JC editors. Adolescents and Adults With Autism Spectrum Disorders. New York, NY: Springer New York (2014). p. 1–13.

[8] Ke F Whalon K Yun J. Social skill interventions for youth and adults with autism spectrum disorder: a systematic review. Rev Educ Res. (2018) 88:3–42. 10.3102/0034654317740334

[9] Humphrey N Symes W. Peer interaction patterns among adolescents with autistic spectrum disorders (ASDs) in mainstream school settings. Autism. (2011) 15:397–419. 10.1177/1362361310387804 21454385

[10] Ratcliffe B Wong M Dossetor D Hayes S. The association between social skills and mental health in school-aged children with autism spectrum disorder, with and without intellectual disability. J Autism Dev Disord. (2015) 45:2487–96. 10.1007/s10803-015-2411-z 25758822

[11] Ellingsen R Bolton C Laugeson E. Evidence-based social skills groups for individuals with autism spectrum disorder across the lifespan. In: Leaf JB editor. Handbook of Social Skills and Autism Spectrum Disorder: Assessment, Curricula, and Intervention. Cham: Springer International Publishing (2017). p. 343–58.

[12] Mendelson JL Gates JA Lerner MD. Friendship in school-age boys with autism spectrum disorders: a meta-analytic summary and developmental, process-based model. Psychol Bull. (2016) 142:601–22. 10.1037/bul0000041 26752425

[13] Baron-Cohen S. Mindblindness: An Essay on Autism and Theory of Mind. Cambridge, MA: MIT Press (1997).

[14] Senju A. Atypical development of spontaneous social cognition in autism spectrum disorders. Brain Dev. (2013) 35:96–101. 10.1016/j.braindev.2012.08.002 22964276

[15] Moody CT Laugeson EA. Social skills training in autism spectrum disorder across the lifespan. Psychiatr Clin N Am. (2020) 43:687–99. 10.1016/j.psc.2020.08.006 33127002

[16] Wright MF Wachs S. Does peer rejection moderate the associations among cyberbullying victimization, depression, and anxiety among adolescents with Autism Spectrum Disorder? Children. (2019) 6:41. 10.3390/children6030041 30836698 PMC6463086

[17] Scott M Milbourn B Falkmer M Black M Bölte S Halladay A. Factors impacting employment for people with autism spectrum disorder: a scoping review. Autism. (2019) 23:869–901. 10.1177/1362361318787789 30073870

[18] Anderson C Butt C Sarsony C. Young adults on the autism spectrum and early employment-related experiences: aspirations and obstacles. J Autism Dev Disord. (2021) 51:88–105. 10.1007/s10803-020-04513-4 32356082

[19] Solomon C. Autism and employment: implications for employers and adults with ASD. J Autism Dev Disord. (2020) 50:4209–17. 10.1007/s10803-020-04537-w 32415532

[20] Howlin P. Social disadvantage and exclusion: adults with autism lag far behind in employment prospects. J Am Acad Child Adolesc Psychiatry. (2013) 52:897–9. 10.1016/j.jaac.2013.06.010 23972691

[21] Cassidy SA Robertson A Townsend E O'Connor RC Rodgers J. Advancing our understanding of self-harm, suicidal thoughts and behaviours in autism. J Autism Dev Disord. (2020) 50:3445–9. 10.1007/s10803-020-04643-9 32880789

[22] Cappadocia MC Weiss JA. Review of social skills training groups for youth with Asperger Syndrome and High Functioning Autism. Res Autism Spectrum Disord. (2011) 5:70–8. 10.1016/j.rasd.2010.04.001

[23] Frankel F Myatt R Feinberg D. Parent-assisted friendship training for children with autism spectrum disorders: effects of psychotropic medication. Child Psychiatry Hum Dev. (2007) 37:337–46. 10.1007/s10578-007-0053-x 17406973

[24] Mandelberg J Frankel F Cunningham T Gorospe C Laugeson EA. Long-term outcomes of parent-assisted social skills intervention for high-functioning children with autism spectrum disorders. Autism. (2014) 18:255–63. 10.1177/1362361312472403 23996903

[25] Reichow B Steiner AM Volkmar F. Social skills groups for people aged 6 to 21 with autism spectrum disorders (ASD). Cochrane Database Syst Rev. (2012) 7:CD008511. 10.1002/14651858.CD008511.pub2 22786515 PMC11975264

[26] Tse J Strulovitch J Tagalakis V Meng L Fombonne E. Social skills training for adolescents with Asperger Syndrome and high-functioning autism. J Autism Dev Disord. (2007) 37:1960–8. 10.1007/s10803-006-0343-3 17216559

[27] Stichter JP Herzog MJ Visovsky K Schmidt C Randolph J Schultz T. Social competence intervention for youth with Asperger Syndrome and high-functioning autism: an initial investigation. J Autism Dev Disord. (2010) 40:1067–79. 10.1007/s10803-010-0959-1 20162344

[28] Webb BJ Miller SP Pierce TB Strawser S Jones WP. Effects of social skill instruction for high-functioning adolescents with Autism Spectrum Disorders. Focus Autism Other Dev Disabil. (2004) 19:53–62. 10.1177/10883576040190010701

[29] Gates JA Kang E Lerner MD. Efficacy of group social skills interventions for youth with autism spectrum disorder: a systematic review and meta-analysis. Clin Psychol Rev. (2017) 52:164–81. 10.1016/j.cpr.2017.01.006 28130983 PMC5358101

[30] Wong C Odom SL Hume KA Cox AW Fettig A Kucharczyk S. Evidence-based practices for children, youth, and young adults with Autism Spectrum Disorder: a comprehensive review. J Autism Dev Disord. (2015) 45:1951–66. 10.1007/s10803-014-2351-z 25578338

[31] Keifer CM Mikami AY Morris JP Libsack EJ Lerner MD. Prediction of social behavior in autism spectrum disorders: explicit versus implicit social cognition. Autism. (2020) 24:1758–72. 10.1177/1362361320922058 32484000 PMC7541482

[32] Wolstencroft J Robinson L Srinivasan R Kerry E Mandy W Skuse D. A systematic review of group social skills interventions, and meta-analysis of outcomes, for children with high functioning ASD. J Autism Dev Disord. (2018) 48:2293–307. 10.1007/s10803-018-3485-1 29423608 PMC5996019

[33] McMahon CM Lerner MD Britton N. Group-based social skills interventions for adolescents with higher-functioning autism spectrum disorder: a review and looking to the future. Adolesc Health Med Ther. (2013) 2013:23–8. 10.2147/AHMT.S25402 23956616 PMC3744120

[34] Gresham FM Elliott SN. Social skills rating system (SSRS). American Guidance Service (1990).

[35] Lerner MD Calhoun CD Mikami AY De Los Reyes A. Understanding parent-child social informant discrepancy in youth with high functioning autism spectrum disorders. J Autism Dev Disord. (2012) 42:2680–92. 10.1007/s10803-012-1525-9 22456819

[36] Lerner MD Mikami AY. A preliminary randomized controlled trial of two social skills interventions for youth with high-functioning Autism Spectrum Disorders. Focus Autism Other Dev Disabil. (2012) 27:147–57. 10.1177/1088357612450613

[37] Bottema-Beutel K Park H Kim SY. Commentary on social skills training curricula for individuals with ASD: social interaction, authenticity, and stigma. J Autism Dev Disord. (2018) 48:953–64. 10.1007/s10803-017-3400-1 29170937

[38] Lerner MD White SW. Moderators and mediators of treatments for youth with autism spectrum disorders. Moderators Mediators Youth Treatment Outcomes. (2015) 146. 10.1093/med:psych/9780199360345.003.0007

[39] Hall LJ Leinert S Jacquez J. A review of social skills manuals for adolescents with Autism Spectrum Disorder. Curr Dev Disord Rep. (2018) 5:77–88. 10.1007/s40474-018-0134-5

[40] McGinnis E Sprafkin RP Gershaw N Klein PC. Skillstreaming the Adolescent: A Guide for Teaching Prosocial Skills. Champaign, IL: Research Press (2012).

[41] Laugeson EA Frankel F. Social Skills for Teenagers With Developmental and Autism Spectrum Disorders: The PEERS Treatment Manual. New York, NY: Routledge (2011). 10.2989/17280583.2013.802447 25860311

[42] Hotton M Coles S. The effectiveness of social skills training groups for individuals with Autism Spectrum Disorder. Rev J Autism Dev Disord. (2016) 3:68–81. 10.1007/s40489-015-0066-533583058

[43] White SW Koenig K Scahill L. Group social skills instruction for adolescents with high-functioning Autism Spectrum Disorders. Focus Autism Other Dev Disabil. (2010) 25:209–19. 10.1177/1088357610380595

[44] Mirzaei SS Pakdaman S Alizadeh E Pouretemad H. A systematic review of program circumstances in training social skills to adolescents with high-functioning autism. Int J Dev Disabil. (2020) 1–10. 10.1080/20473869.2020.1748802 PMC9122374 35603001

[45] Zheng S Kim H Salzman E Ankenman K Bent S. Improving social knowledge and skills among adolescents with autism: systematic review and meta-analysis of UCLA PEERS® for adolescents. J Autism Dev Disord. (2021) 1–16. 10.1007/s10803-021-04885-1 33512626

[46] Laugeson EA Park MN. Using a CBT approach to teach social skills to adolescents with autism spectrum disorder and other social challenges: the PEERS® method. J Ration Emotive Cogn Behav Therapy. (2014) 32:84–97. 10.1007/s10942-014-0181-8

[47] Gresham FM Watson TS Skinner CH. Functional behavioral assessment: principles, procedures, and future directions. School Psychol Rev. (2001) 30:156–72. 10.1080/02796015.2001.12086106

[48] Prata J Lawson W Coelho R. Parent training for parents of children on the autism spectrum: a review. Health. (2018) 5:3–8. 10.21035/ijcnmh.2018.5.3

[49] Corona LL Janicki C Milgramm A Christodulu KV. Brief Report: reductions in parenting stress in the context of PEERS-A social skills intervention for adolescents with Autism Spectrum Disorder. J Autism Dev Dis. (2019) 49:5073–7. 10.1007/s10803-019-04201-y 31473951

[50] Rabin SJ Laugeson EA Mor-Snir I Golan O. An Israeli RCT of PEERS®: intervention effectiveness and the predictive value of parental sensitivity. J Clin Child Adolesc Psychol. (2020) 1–17. 10.1080/15374416.2020.1796681 32780594

[51] Shum KK-M Cho WK Lam LMO Laugeson EA Wong WS Law LSK. Learning how to make friends for chinese adolescents with autism spectrum disorder: a randomized controlled trial of the Hong Kong Chinese Version of the PEERS® intervention. J Autism Dev Disord. (2019) 49:527–41. 10.1007/s10803-018-3728-1 30143950

[52] Hong JK Oh M Bong G Kim J-H Bahn G Cho I-H. Age as a moderator of social skills intervention response among korean adolescents with Autism Spectrum Disorder. J Autism Dev Disord. (2019) 49:1626–37. 10.1007/s10803-018-3859-4 30547257

[53] Yamada T Miura Y Oi M Akatsuka N Tanaka K Tsukidate N. Examining the treatment efficacy of PEERS in Japan: improving social skills among adolescents with autism spectrum disorder. J Autism Dev Disord. (2020) 50:976–97. 10.1007/s10803-019-04325-1 31823217 PMC7010628

[54] van Pelt BJ Idris S Jagersma G Duvekot J Maras A van der Ende J. The ACCEPT-study: design of an RCT with an active treatment control condition to study the effectiveness of the Dutch version of PEERS® for adolescents with autism spectrum disorder. BMC Psychiatry. (2020) 20:274. 10.1186/s12888-020-02650-9 32487179 PMC7268391

[55] Laugeson EA Gantman A Kapp SK Orenski K Ellingsen R. A randomized controlled trial to improve social skills in young adults with Autism Spectrum Disorder: the UCLA PEERS® program. J Autism Dev Disord. (2015) 45:3978–89. 10.1007/s10803-015-2504-8 26109247

[56] Callenmark B Kjellin L Rönnqvist L Bölte S. Explicit versus implicit social cognition testing in autism spectrum disorder. Autism. (2014) 18:684–93. 10.1177/1362361313492393 24104519 PMC4230543

[57] Hull L Petrides KV Allison C Smith P Baron-Cohen S Lai M-C. “Putting on My Best Normal”: social camouflaging in adults with autism spectrum conditions. J Autism Dev Disord. (2017) 47:2519–34. 10.1007/s10803-017-3166-5 28527095 PMC5509825

[58] Fombonne E. Camouflage and autism. J Child Psychol Psychiatry Allied Discipl. (2020) 61:735–8. 10.1111/jcpp.13296 32658354

[59] Care DoHS. ‘Right To Be Heard': The Government's Response to the Consultation on Learning Disability and Autism Training for Health and Care Staff (2019).

